# Disentangling the Effects of Suicide Attempts and Psychiatric Diagnosis Based on a Genotype-Informed Dynamic Model of the Serotonin Presynapse

**DOI:** 10.3390/genes16101141

**Published:** 2025-09-26

**Authors:** Lana Radenković, Maja Pantović-Stefanović, Goran Brajušković, Maja Ivković, Dušanka Savić-Pavićević, Jovan Pešović

**Affiliations:** 1 University of Belgrade-Faculty of Biology, Centre for Human Molecular Genetics, Studentski Trg 16, 11000 Belgrade, Serbia; lana.radenkovic@bio.bg.ac.rs (L.R.); brajuskovic@bio.bg.ac.rs (G.B.); duska@bio.bg.ac.rs (D.S.-P.); 2 Clinic for Psychiatry, University Clinical Centre of Serbia, Pasterova 2, 11000 Belgrade, Serbia; majapantovic@yahoo.it (M.P.-S.); majaivkovic123@gmail.com (M.I.); 3 University of Belgrade-Faculty of Medicine, Doktora Subotića 8, 11000 Belgrade, Serbia

**Keywords:** dynamic model, serotonin system, genetic variants, suicide attempt, bipolar disorder, major depressive disorder, schizophrenia

## Abstract

Background: Suicide attempts often co-occur with bipolar disorder (BD), major depressive disorder (MDD), and schizophrenia (SCH). Although impairments of the serotonin (5-HT) system have been associated with suicide attempts, it remains unclear whether these alterations reflect suicidal behavior or are confounded by underlying psychiatric diagnosis. This study used a genotype-informed dynamic model of the 5-HT presynapse to disentangle the effects of suicide attempts and psychiatric diagnosis. Methods: We applied a personalized dynamic model of the 5-HT presynapse to 392 psychiatric patients (with BD, MDD, or SCH), categorized by suicide attempt status, and 140 unaffected individuals. The model incorporated five variants across *TPH2*, *SLC6A4*, and *MAOA* genes simulating individual-specific concentration changes of five 5-HT-related molecular species. Model outputs were summarized by six statistical measures (mean, median, maximum, standard deviation, skewness, and kurtosis) and compared across groups. Results: No significant differences were found across groups defined by suicide attempt status and unaffected individuals. However, diagnosis significantly influenced 5-hydroxyindoleacetic acid (5-HIAA) mean, median, maximum, and standard deviation (all *p* < 0.05). BD patients had lower 5-HIAA levels than SCH patients (mean: *p* = 0.013; median: *p* = 0.013; maximum: *p* = 0.014; standard deviation: *p* = 0.014). MDD patients also showed lower 5-HIAA levels than SCH patients for the same measures, with differences approaching significance. No significant difference was observed between BD and MDD patients. A diagnosis-by-suicide attempt status interaction was observed for 5-HIAA skewness (*p* = 0.013). Conclusions: Model-derived 5-HT profiles were shaped primarily by diagnosis, while temporal dynamics of 5-HIAA, rather than its absolute levels, was associated with suicide attempt status. Thus, personalized dynamic modeling incorporating genetic variants may aid in detecting subtle molecular signatures across diagnoses and suicidal behavior.

## 1. Introduction

Suicidal behavior is a major public health concern, ranking as the second leading cause of death among adolescents and young adults (Suicide Prevention: Facts About Suicide. Centers for Disease Control and Prevention; 2023). It is a complex and diverse condition that includes suicidal ideation, suicide attempt, and death by suicide. A deeper understanding of the biology and pathology underlying suicidal behavior is essential for developing effective prevention strategies and remains a critical public health priority. Current models suggest that suicide attempts and completed suicides result from interactions between individual vulnerabilities and external stressors [1]. At the individual level, triggers may include recent stressful life events and the presence of psychiatric conditions. Mental health disorders, particularly bipolar disorder (BD), major depressive disorder (MDD), and schizophrenia (SCH), are strongly associated with increased risk of suicide attempt. Individuals with these conditions have been shown to have a suicide mortality rate approximately ten times higher than that of the general population [2]. Evidence from family, twin, and adoption studies suggests a genetic contribution to suicidal behavior, and its interaction with environmental factors [3,4]. Individuals with a family history of suicidal behavior have approximately five times higher risk [3]. Twin studies show greater concordance in identical twins, with heritability estimates up to 45%, while adoption studies reveal a sevenfold increased risk of completed suicide among biological relatives [3].

Post-mortem studies of individuals who died by suicide have revealed consistent alterations in the serotonin (5-HT) system [5,6,7,8]. These include reduced numbers of 5-HT transporter sites, changes in the number and structure of 5-HT-producing neurons in the brainstem, lower binding affinity of 5-HT transporters and receptors, and decreased 5-HT responsiveness [9,10,11,12]. Additionally, reduced levels of the primary metabolic product of 5-HT, 5-hydroxyindoleacetic acid (5-HIAA), have been found in the cerebrospinal fluid (CSF) of suicide victims [9,10,11,13,14,15]. However, due to the design of these studies, it is difficult to determine whether the observed changes are associated with suicide itself or with the underlying psychiatric disorders.

Multiple genetic variants within 5-HT system genes have been associated with suicidal behavior through candidate gene studies [1,6,16]. Recent genetic studies, including genome-wide association studies (GWAS) [17,18,19,20] and Mendelian randomization analyses [21,22,23], provide complementary insights into the genetic pathways influencing psychiatric disorders and suicide attempt risk. Nevertheless, findings from these studies have been inconsistent, largely due to underpowered studies, the reliance of GWAS on statistical associations rather than underlying biological mechanisms, and the oversight of interactions among genetic variants and processes in biological systems.

In our previous work, we developed a computational dynamic model of the 5-HT presynapse that simulates the synthesis, reuptake, and degradation of 5-HT based on individual genetic variants in key 5-HT system genes: *TPH2*, *SLC6A4* and *MAOA* [24]. The model captures dynamic changes in molecular concentrations throughout the simulation, taking into account the interactions between the elements of the 5-HT presynapse as well as the individual’s genotype-based modulation of enzyme and transporter activity. The model initiates with the uptake of dietary tryptophan into the presynaptic neuron, which is converted to 5-hydroxytryptophan (5-HTP) by the rate-limiting enzyme tryptophan hydroxylase 2 (TPH2), and then further processed into free intracellular serotonin (fc5-HT). The fc5-HT is stored in vesicles as vesicular 5-HT (v5-HT), released into the synaptic cleft as extracellular 5-HT (e5-HT), and either degraded or reabsorbed via the serotonin transporter (SERT, encoded by the *SLC6A4* gene). Finally, monoamine oxidase A (MAOA) catalyzes the degradation of fc5-HT into 5-HIAA. The model produces a set of time series that capture the changes in the concentrations of 5-HTP, fc5-HT, v5-HT, e5-HT and 5-HIAA, which are referred to as molecular species [25]. The predicted profiles of these molecular species vary across individuals, depending on their specific genotype combinations. The model has been validated on 140 unaffected individuals and applied to a cohort of patients diagnosed with BD and categorized according to suicide attempt status [24]. We observed lower predicted mean 5-HIAA levels in BD patients with a history of suicide attempt compared to unaffected individuals.

In the present study, we extend the application of our dynamic model of 5-HT presynapse by including individuals diagnosed with MDD and SCH, in addition to BD, both with and without a history of suicide attempt. Our aim was to assess whether model-derived differences in 5-HT-related molecular species reflect suicide attempt status or are more closely associated with psychiatric diagnosis.

## 2. Materials and Methods

### 2.1. Study Participants

**Figure 1:**
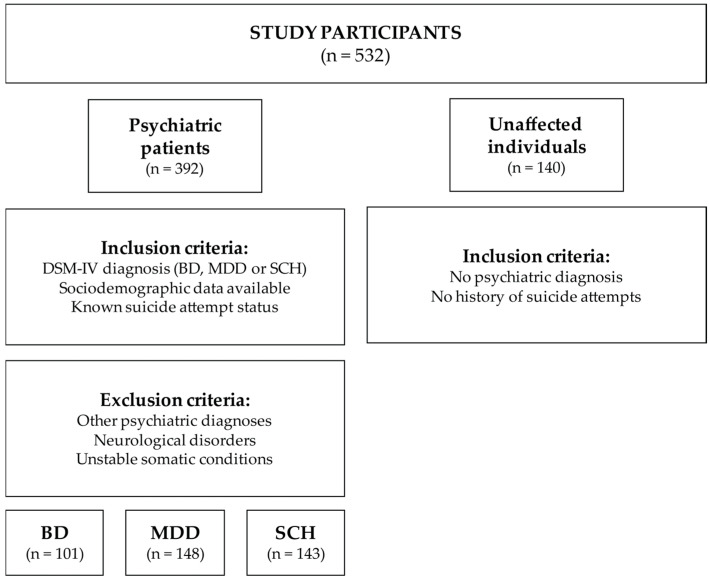
Flow diagram of participant inclusion and exclusion criteria.

The study included 392 unrelated psychiatric patients diagnosed with BD (*n* = 101, 25.77%), MDD (*n* = 148, 37.76%) or SCH (*n* = 143, 36.48%) using the Structured Clinical Interview for DSM-IV Axis I Disorders [26], described in our previous study [27]. Additionally, 140 unaffected individuals were examined using the same interview. The patients were recruited after five weeks of hospitalization at the Department of Psychiatry, University Clinical Centre of Serbia, between 2006 and 2016. Inclusion criteria were a confirmed DSM-IV diagnosis, sociodemographic data (sex and age), and a history of suicide attempts. Exclusion criteria included the presence of other psychiatric disorders, neurological disorders, or unstable somatic conditions (Figure 1). Patients were categorized according to suicide attempt status. Patients with a history of suicide attempt (SA, *n* = 175, 44.64%) were hospitalized following an attempt, while the patients without a history of suicide attempt (non-SA, *n* = 217, 55.36%) were hospitalized due to recurrence of their primary psychiatric disorder and without a history of suicide attempts. All participants provided written informed consent. Ethical approval was obtained from the Ethics Committee of the University Clinical Centre of Serbia (Decision no. 340/4; 21 July 2021). The study was conducted in accordance with the Declaration of Helsinki.

### 2.2. Genotyping

Peripheral blood samples from patients with MDD and SCH were collected and genotyped, as described in detail in Radenkovic et al. [24]. We selected five genetic variants known to influence 5-HT metabolism: three single-nucleotide polymorphisms in the *TPH2* gene (rs111798998, rs4290270, rs7305115), the 5-HT transporter-linked polymorphic region (5-HTTLPR) in the *SLC6A4* gene, and the upstream variable number of tandem repeats (uVNTR) in the *MAOA* gene [28,29,30,31,32]. Chromosomal locations and allele frequencies for each variant in the general population are provided in Table S1. Genotype data for BD patients and unaffected individuals were obtained from our previous study [24]. Approximately 10% of all DNA samples were randomly selected for duplicate analysis. The results showed complete concordance.

### 2.3. Dynamic 5-HT Presynapse Model Simulation and Extraction of Model-Derived Features

A detailed description of the developed model of 5-HT presynapse, including its structure, parameterization, and the integration of genotype-dependent effects, is provided in Radenkovic et al. [24]. Briefly, the model consists of eight differential equations that quantitatively represent reactions catalyzed by TPH2, SERT and MAOA. We incorporated the coefficient C_genotype_ which adjusts the activity of these proteins based on individual genotypes the functionally relevant variants: *TPH2* (rs111798998, rs4290270, rs7305115), *SLC6A4* (5-HTTLPR), and *MAOA* (uVNTR). These variants modulate gene expression in an allele-specific manner, influencing mRNA levels, as measured through allelic imbalance and luciferase reporter assays [28,29,30,31,32], and thereby affecting 5-HT synthesis, reuptake, and degradation. Specifically, rs111798998 G and rs7305115 A increase *TPH2* mRNA expression ~3- and 1.7-fold, respectively, while rs4290270 A decreases expression ~1.4-fold. The *SLC6A4* 5-HTTLPR L allele increases transporter mRNA ~3-fold, and the *MAOA* uVNTR 4R and 3.5R alleles increase *MAOA* mRNA 5–6-fold. These genetically determined differences in enzyme/transporter expression are incorporated into the model as variations in protein abundance, thereby altering the concentrations and temporal dynamics of 5-HT–related molecular species. This in turn allowed for the simulation of individual-specific 5-HT dynamics and the calculation of the concentrations of 5-HT-related molecular species: 5-HTP, fc5-HT, v5-HT, e5-HT, and 5-HIAA. We applied this model of 5-HT presynapse to MDD and SCH patient groups, while the simulation data for BD patients and unaffected individuals were retrieved from our previous work [24].

Simulation of the model resulted in an output consisting of raw time series data for each of the five molecular species, simulated for every study participant. Given that direct comparisons between individuals or diagnostic groups were not possible due to the complexity of the raw time series data, we extracted a set of descriptive statistical measures for each molecular species and each participant. Specifically, we summarized the time series data by calculating the mean, median, standard deviation, maximum, skewness and kurtosis. These features were selected based on their ability to represent different aspects of time series data, as shown by Guo et al. [33]. Mean and median values represent measures of central tendency and reflect the average concentrations of molecular species over the course of the simulation. Standard deviation and maximum values reflect the dispersion and range of the time series data and capture its variability and peak levels, respectively. Skewness (degree of asymmetry in the distribution) and kurtosis (degree of peakedness and presence of outliers) represent the shape of the time series and provide important supplementary information for a comprehensive characterization of the entire time series. By extracting these six statistical measures for each of the five molecular species (5-HTP, fc5-HT, v5-HT, e5-HT, and 5-HIAA), we obtained a set of 30 model-derived features that was used for subsequent statistical analyses.

### 2.4. Statistical Analysis

All statistical analyses were conducted using R (v.4.1.0) and Python3 (v.3.11.4). The distributions of age and model-derived features were assessed for normality using Q–Q plots and the Shapiro–Wilk test. Differences in age between SA and non-SA patients were evaluated using the Mann–Whitney U test, while comparisons involving both patient groups and unaffected individuals were conducted using the Kruskal–Wallis test. Differences in sex distribution were assessed using the chi-square test of independence. All model-derived features were compared between SA and non-SA patients, and unaffected individuals using the Kruskal–Wallis test.

To examine the joint association of suicide attempt status and psychiatric diagnosis (BD, MDD, and SCH) on model-derived features, we used the Aligned Rank Transform (ART) ANOVA, a nonparametric statistical test that allows factor analysis of data that do not meet normality assumptions. It was implemented via the ARTool package in R (v.0.11.2) [34], https://github.com/mjskay/ARTool (accessed on 21 May 2025). In our analysis, suicide attempt status (yes/no) and psychiatric diagnosis were entered as fixed between-subjects factors. The ART ANOVA was performed separately for all model-derived features. We tested for main effects of suicide attempt status and diagnostic group, as well as for their interaction effects, which indicate whether the relationship between suicide attempt status and a model feature varied across diagnostic groups. Statistical significance was set at *p* < 0.05, and *p*-values between 0.05 and 0.10 were interpreted as trends.

## 3. Results

### 3.1. Overview of Study Participants’ Data

Our study included 392 unrelated psychiatric patients diagnosed with BD (*n* = 101, 25.77%), MDD (*n* = 148, 37.76%), or SCH (*n* = 143, 36.48%). Approximately 45% of the patients had a history of suicide attempts. Additionally, a previously described cohort of 140 unaffected individuals was included as a control group [24]. The demographic characteristics of study participants are shown in Table 1. There were no significant differences in age (Mann–Whitney U test: U = 20,007.00, *p* = 0.361) or sex (Chi-square test: χ^2^ = 0.25, *p* = 0.619) between SA and non-SA patients. A comparison of age (Kruskal–Wallis test: H = 4.76, *p* = 0.093) and sex (Chi-square test: χ^2^ = 0.43, *p* = 0.805) across all three groups (SA, non-SA patients and unaffected individuals) also revealed no significant differences.

### 3.2. 5-HT Dynamic Model Simulation Results

Allele and genotype frequencies for the analyzed *TPH2* variants (rs111798998, rs4290270, and rs7305115), the *SLC6A4* variant 5-HTTLPR, and *MAOA* uVNTR variant in all study participants are given in Table S2. For each participant, we obtained the time-dependent output of the computational 5-HT presynapse model [24] showing the changes in concentrations of 5-HTP, fc5-HT, v5-HT, e5-HT, and 5-HIAA over the simulation period. Next, each time series was summarized using the statistical measures mean, median, standard deviation, maximum, skewness and kurtosis. The mean values of these measures for all groups stratified by diagnosis and suicide attempt status are given in Table S3.

**Figure 2:**
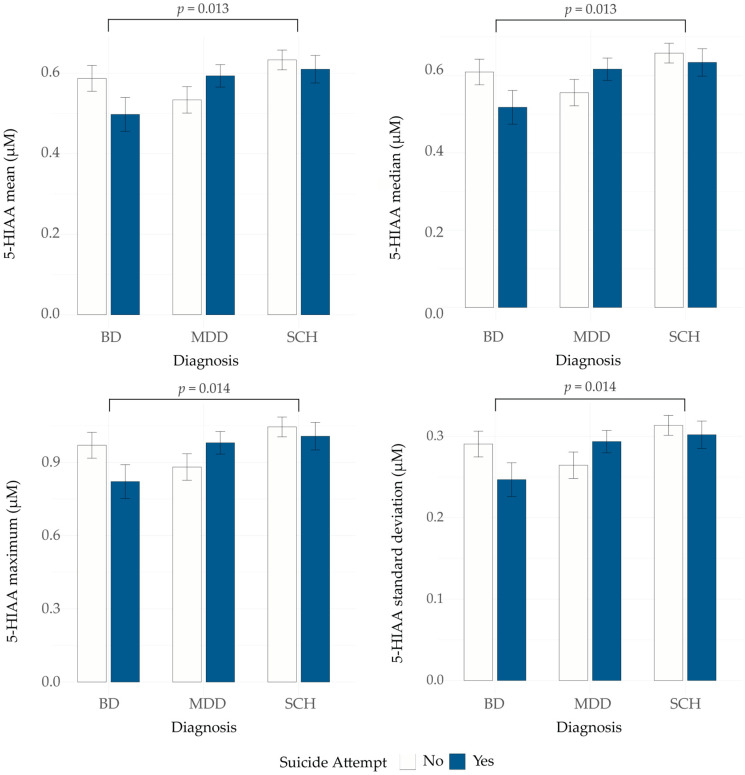
5-hydroxyindoleacetic acid (5-HIAA) mean, median, maximum and standard deviation in patients with BD, MDD and SCH, with and without a history of suicide attempt. BD—bipolar disorder; MDD—major depressive disorder; SCH—schizophrenia.

To evaluate whether the 5-HT presynapse model-derived measures reflect suicide attempt status, we compared their values between three groups: all SA patients, all non-SA patients and unaffected individuals. No statistically significant differences were observed (Kruskal–Wallis test, all *p* > 0.05, Table 2). Next, we examined the potential interaction between suicide attempt status and psychiatric diagnosis in the patient group. A two-way ART ANOVA was performed with psychiatric diagnosis and suicide attempt status as factors to examine both their main and interaction effects on model-derived measures. The analysis revealed no significant main effects of suicide attempt status across any of the measures (all *p*-values > 0.05; Table S4). We observed a significant main effect of psychiatric diagnosis on four 5-HIAA-related measures: mean (F = 4.61, *p* = 0.011), median (F = 4.54, *p* = 0.011), maximum (F = 4.62, *p* = 0.010) and standard deviation (F = 4.49, *p* = 0.012). Post hoc comparisons revealed that individuals with BD had significantly lower 5-HIAA values across these four measures compared to individuals with SCH (mean: *p* = 0.013; median: *p* = 0.013; maximum: *p* = 0.014; standard deviation: *p* = 0.014) (Figure 1). MDD patients also showed lower values compared to SCH patients for the same 5-HIAA measures, with differences approaching statistical significance (mean: *p* = 0.060; median: *p* = 0.070; maximum: *p* = 0.054; standard deviation: *p* = 0.066). Differences between BD and MDD were not significant (all *p* > 0.05). This may be due to the observed opposite pattern of 5-HIAA mean, median, maximum, and standard deviation between SA and non-SA patients in MDD and BD groups (Figure 2 and Table S4).

**Figure 3:**
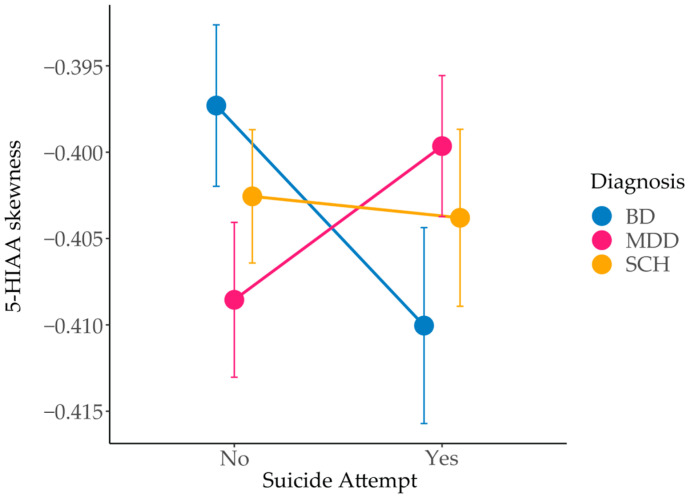
Interaction effect of diagnosis and suicide attempt status on 5-hydroxyindoleacetic acid (5-HIAA) skewness in patients with BD, MDD and SCH, with and without a history of suicide attempt. BD—bipolar disorder; MDD—major depressive disorder; SCH—schizophrenia.

In addition to the main effects of diagnosis on 5-HIAA measures, we observed a significant interaction between diagnosis and suicide attempt status for 5-HIAA skewness (F = 4.38, *p* = 0.013) (Figure 3). Moreover, several other model-derived measures showed trends toward an interaction between diagnosis and suicide attempt status, including fc5-HT (mean: *p* = 0.064; median: *p* = 0.084; maximum: *p* = 0.057; standard deviation: *p* = 0.065), v5-HT (mean: *p* = 0.067; maximum: *p* = 0.054; standard deviation: *p* = 0.063), e5-HT (median: *p* = 0.087), and 5-HIAA (mean: *p* = 0.087; maximum: *p* = 0.074; kurtosis: *p* = 0.099) (Table S4).

Taken together, these findings suggest that the 5-HT model-derived features predominantly reflect psychiatric diagnosis, as significant differences were observed primarily between the diagnostic groups. Although suicide attempt status alone was not a statistically significant factor, its influence appears to be diagnosis-specific.

## 4. Discussion

In the present study, we used a previously developed dynamic model of 5-HT presynapse to study 5-HT dynamics beyond conventional genetic associations, since the model integrates functionally validated variants with experimentally demonstrated effects on *TPH2*, *SLC6A4*, and *MAOA* expression into a mechanistic framework [24].

We applied the model to a large, diagnostically diverse cohort, including individuals with BD, MDD, SCH, and unaffected individuals, and showed that model-derived features were associated primarily with diagnosis, rather than suicide attempt status. Furthermore, we did not observe significant differences in age or sex between patients stratified by suicide attempt status.

Among five molecular species derived from the model, 5-HIAA emerged as the most informative for differentiating diagnostic groups. Specifically, we observed a significant main effect of diagnosis on four measures of 5-HIAA dynamics—mean, median, maximum, and standard deviation, suggesting their primary association with psychiatric diagnosis. BD patients exhibited significantly reduced levels of these measures compared to SCH, consistent with prior findings [6,16,24,35,36]. Moreover, we observed a trend of lower levels of the same 5-HIAA measures in MDD patients. Interestingly, MDD patients demonstrated an opposite pattern when stratified by suicide attempt status compared to the corresponding groups in BD and SCH patients, which aligns with the inconsistent findings reported in previous studies. Some studies observed reduced 5-HIAA levels in drug-naïve patients or after SSRI treatment [37,38], whereas others reported elevated 5-HIAA levels in specific subgroups [39,40]. These findings suggest that serotonergic profiles in MDD are heterogeneous and may be influenced by other moderating factors including medication status, symptom severity, and underlying neurobiological differences [41].

Furthermore, large-scale genetic studies, including GWAS and Mendelian randomization analyses, highlight a substantial shared genetic architecture between SCH and BD which only partially overlaps with MDD [23], supporting our observation that MDD differs from BD and SCH in relation to suicidal behavior [21,22,23]. Specifically, the genetic architecture of MDD correlates strongly with self-harm, including suicide attempt, but shows a weaker association with completed suicide [21]. Moreover, genetic effects of MDD can act both directly and indirectly through intermediate phenotypes such as insomnia [22], which itself is associated with increased suicide risk. Notably, serotonergic signaling through 5-HT receptors plays an important role in sleep regulation, suggesting a potential relationship between 5-HT dysregulation, sleep disturbances, and suicide attempts. Taken together, these findings indicate that the genetic architecture of MDD likely reflects both direct and indirect genetic influences on 5-HT pathways in the presynapse.

Analysis of 5-HIAA time-series skewness offers further insight into temporal dynamics of 5-HT metabolism in the presynapse. While the calculated mean, median, and maximum values reflect overall quantity of 5-HIAA and can be directly compared to empirical measurements, skewness reflects the timing of its accumulation in the system. In our model, 5-HIAA removal is held constant across individuals, while synthesis varies depending on genotype [24]. Consequently, the skewness of the 5-HIAA concentration time profile primarily reflects the synthesis rate. A more negative skew indicates a delayed accumulation, with concentration rising gradually and peaking later, whereas a less negative skew corresponds to earlier peak levels. We observed a significant interaction between diagnosis and suicide attempt status for 5-HIAA skewness (*p* = 0.013), suggesting that the timing of its accumulation may differ depending on both clinical diagnosis and history of suicide attempts.

Within each diagnostic group, subtle differences in 5-HIAA skewness were observed between patients grouped by suicide attempt status. MDD SA patients had on average slightly earlier 5-HIAA peaks (skewness –0.40) compared to non-SA (skewness –0.41), whereas BD patients showed the opposite pattern (skewness –0.41 in SA versus –0.40 in non-SA patients). Nevertheless, although these differences are modest, they reflect nuanced shifts in the timing of 5-HT metabolism, which may influence its accumulation, turnover, and long-term depletion, with potential implications for vulnerability to suicide attempt. Earlier 5-HIAA peaks combined with higher 5-HIAA concentrations suggest higher 5-HT turnover in MDD. This is consistent with reports of elevated turnover in unmedicated MDD patients [40], MDD patients with comorbid panic disorder [42], and with findings of higher 5-HIAA levels in MDD compared to BD in acute phase of the disorder [36]. Conversely, our results suggest lower 5-HT turnover in BD, in line with studies showing reduced 5-HIAA measurements during depressive and manic episodes compared to MDD and healthy controls [36], and with decreased CSF 5-HIAA in BD patients with a history of suicide attempts [43,44]. SCH patients exhibited minimal variation, with skewness values remaining close to –0.40 regardless of suicide attempt status, suggesting more stable serotonergic dynamics and supporting the view that 5-HT dysfunction may play a less central role in SCH pathophysiology [45,46]. To our knowledge, direct published measurements of the 5-HT degradation speed in psychiatric conditions appear limited.

By incorporating genetic variants in *TPH2*, *SLC6A4*, and *MAOA* genes, our model captures individual differences in 5-HT synthesis, reuptake, and degradation. The results reveal distinct serotonergic patterns associated with diagnosis, suicide attempt status, and their interaction, highlighting complex, diagnosis-specific alterations linked to suicidality. Furthermore, an overlap exists with other traits, such as genetic liability to substance use, alcohol abuse, insomnia, and neurodevelopmental disorders including ADHD, OCD, and autism [21,22,23]. This suggests that genetic liability for major psychiatric disorders may influence suicidal behaviors through both disorder-specific and shared mechanisms. Traditional group-level analyses often overlook important inter-individual variability in psychiatric disorders. In contrast, our personalized, genotype-informed simulations detect subtle, diagnosis-specific 5-HT dynamics related to suicidal behavior. Together, these results demonstrate that stratifying individuals by both genotype and diagnosis provides deeper insight into the serotonergic mechanisms of suicidality, offering a promising path toward personalized psychiatry and improved risk assessment. Evidence from functional and structural neuroimaging studies supports this implication. Shared disruptions in the cerebellum, thalamus, hippocampus, and cortical areas were observed in MDD and SCH, alongside diagnosis-specific alterations in the prefrontal cortex, amygdala, and temporal poles [47]. Structural changes have also been reported in individuals with a history of suicide attempt, including reduced cortical thickness [48], decreased brain volume [49], and increased activity in HTR1A-enriched regions such as the right superior temporal gyrus [50]. These findings suggest that subtle, diagnosis-specific shifts in 5-HT metabolism may have functional consequences in neural circuits involved in executive function and suicidal behavior.

Although our model incorporates kinetic parameters from the experimental literature and was validated both by comparison with other published models and by comparing simulated levels of 5-HT molecular species to published experimental measurements in a cohort of 140 unaffected individuals [24], several limitations of the study should be acknowledged. First, the model focuses exclusively on 5-HT pathways and a small set of functional genetic variants, which cannot capture the full genetic architecture influencing suicide risk and psychiatric diagnoses. Due to this, we were unable to capture the effect of gene–environment interactions or the effects of other neurotransmitter systems, such as the dopamine system. The existing model could be extended to include additional genes and variants within the 5-HT pathway, assuming well-characterized functional parameters for each variant, supported by experimental evidence. This could make the model more biologically realistic and potentially more precise by capturing a broader spectrum of genetic influences. However, incorporating multiple genes further increases mathematical complexity, computational requirements, and the likelihood of non-linear interactions, which could make the system more difficult to interpret and more challenging to validate against empirical data. Moreover, the individual effects of the selected genetic variants are inherently small, and the resulting changes in molecular species concentrations are correspondingly modest. Although these small alterations might appear negligible in magnitude, they are biologically relevant given the high sensitivity of 5-HT signaling pathways. Such fine-scale modulation can have significant downstream effects on receptor activation, neuronal excitability, and network dynamics. Nevertheless, detecting and interpreting these subtle genotype-driven differences remain challenging, particularly in the context of complex, multifactorial psychiatric disorders. Furthermore, our sample size limited the statistical power to detect subtle effects associated with phenotypic subgroups and strata, as well as the effects of the disease duration, prescribed medication, or means of suicide attempt.

Future work should extend the model to include additional genetic variants in serotonergic and related pathways and should integrate longitudinal clinical data, such as mood fluctuations, medication status, and behavioral outcomes. Applying the model in larger patient cohorts may help to further elucidate robust interactions between the elements of the 5-HT presynapse and improve its predictive utility for suicide risk stratification.

## Figures and Tables

**Table 1:** Demographic data of study participants.

	BD *n* = 101		MDD *n* = 148		SCH *n* = 143		Total *n* = 392			Unaffected Individuals	
	SA *n* = 46	non-SA *n* = 55	SA *n* = 76	non-SA *n* = 72	SA *n* = 53	non-SA *n* = 90	SA *n* = 175	non-SA *n* = 217	p-value ^1^	*n* = 140	*p*-value ^2^
Age ^3^ ± SD	43.74 ± 11.7	39.05 ± 10.9	47.91 ± 9.7	50.99 ± 8.2	31.11 ± 8.7	35.47 ± 9.5	42.63 ± 11.6	41.53 ± 11.7	0.361 ^4^	40.34 ± 11.19	0.093 ^5^
Sex (*n*, %)											
female	34 (73.9%)	43 (78.2%)	55 (72.4%)	44 (61.1%)	29 (54.7%)	53 (58.9%)	118 (67.4%)	140 (64.5%)	0.619 ^6^	48 (34.29%)	0.805 ^6^
male	12 (26.1%)	12 (21.8%)	21 (27.6%)	28 (38.9%)	24 (45.3%)	37 (41.1%)	57 (32.6%)	77 (35.5%)		92 (65.71%)	

BD—bipolar disorder; MDD—major depressive disorder; SCH—schizophrenia; SA—patients with a history of suicide attempt; non-SA—patients without a history of suicide attempt. ^1^—Comparison between patients with and without suicide attempts; ^2^—Comparison between unaffected individuals, patients with and without suicide attempts; ^3^—Age is given in years; ^4^—Mann–Whitney U test; ^5^—Kruskal–Wallis test; ^6^—Pearson’s χ^2^ test.

**Table 2:** Average values of model-derived statistical measures in unaffected individuals, patients with and without a history of suicide attempts.

		Unaffected Individuals *n* = 140	SA *n* = 175	Non-SA *n* = 217	*p*-Value ^1^
5-HTP	mean	13.63	13.67	13.65	0.641
	median	6.55	6.58	6.57	0.635
	maximum	42.24	42.36	42.32	0.549
	standard deviation	14.58	14.63	14.61	0.583
	skewness	0.79	0.79	0.79	0.637
	kurtosis	2.09	2.09	2.09	0.624
fc5-HT	mean	0.89	0.90	0.89	0.348
	median	0.57	0.58	0.57	0.337
	maximum	2.40	2.43	2.42	0.375
	standard deviation	0.83	0.84	0.84	0.363
	skewness	0.61	0.60	0.60	0.523
	kurtosis	1.85	1.84	1.85	0.567
v5-HT	mean	2.49	2.52	2.51	0.347
	median	1.69	1.71	1.71	0.489
	maximum	6.50	6.55	6.53	0.376
	standard deviation	2.26	2.28	2.28	0.345
	skewness	0.55	0.54	0.55	0.432
	kurtosis	1.77	1.77	1.77	0.558
e5-HT	mean	0.07	0.07	0.07	0.066
	median	0.04	0.04	0.04	0.415
	maximum	0.20	0.20	0.20	0.273
	standard deviation	0.07	0.07	0.07	0.475
	skewness	0.65	0.65	0.65	0.533
	kurtosis	1.90	1.89	1.90	0.554
5-HIAA	mean	0.61	0.57	0.59	0.725
	median	0.64	0.60	0.61	0.771
	maximum	1.01	0.95	0.97	0.753
	standard deviation	0.30	0.28	0.29	0.704
	skewness	−0.40	−0.40	−0.40	0.537
	kurtosis	2.11	2.11	2.11	0.353

SA—patients with a history of suicide attempt; non-SA—patients without a history of suicide attempt; 5-HTP—5-hydroxytryptophan; fc5-HT—free cellular serotonin (5-HT); v5-HT—vesicular 5-HT; e5-HT—extracellular 5-HT; 5-HIAA—5-hydroxyindoleacetic acid. ^1^—Kruskal–Wallis test. Mean, median, maximum, and standard deviation are in given in μM units; skewness and kurtosis are unitless.

## Data Availability

The datasets generated during the current study that includes patient genotype information, is not publicly available due to privacy concerns regarding personal genetic data. These datasets are available from the corresponding author upon reasonable request.

## Supplementary Materials

The following supporting information can be downloaded at: https://www.mdpi.com/article/10.3390/genes16101141/s1, Table S1: Selected genetic variants in serotonin system genes and allele frequencies.; Table S2: Allele and genotype frequencies in *TPH2*, *SLC6A4*, and *MAOA* genetic variants in patients with bipolar disorder, major depressive disorder, schizophrenia and unaffected individuals.; Table S3: Average values of model-derived statistical measures in patients with bipolar disorder, major depressive disorder, schizophrenia and unaffected individuals.; Table S4: Aligned Rank Transform (ART) ANOVA results for all model-derived features across diagnostic and suicide attempt groups [29,51,52,53].

## Abbreviations

The following abbreviations are used in this manuscript:

BD	Bipolar disorder
MDD	Major depressive disorder
SCH	Schizophrenia
5-HT	Serotonin
5-HIAA	Serotonin degradation product 5-hydroxy-3-indolacetic acid
CSF	Cerebrospinal fluid
GWAS	Genome wide association study
5-HTP	Serotonin precursor 5-hydroxytryptophan
TPH2	Tryptophan hydroxylase 2
fc-5HT	Free cellular serotonin
v5-HT	Serotonin stored in the synaptic vesicle
e5-HT	Extracellular serotonin
SERT	Serotonin transporter
MAOA	Monoamine oxidase A
SA	Patients with a history of suicide attempt
non-SA	Patients without a history of suicide attempt
5-HTTLPR	5-HT transporter linked polymorphic region
uVNTR	Upstream variable number tandem repeat
ART	Aligned rank transform
