# Supplementary material for: Disentangling the Effects of Suicide Attempts and Psychiatric Diagnosis Based on a Genotype-Informed Dynamic Model of the Serotonin Presynapse
Source: Genes (Basel). 2025 Sep 26;16(10):1141. doi: 10.3390/genes16101141 (PMC12563504; doi:10.3390/genes16101141)
Supplement: Supplementary file 1 [file genes-16-01141-s001.zip › genes-3858126-supplementary.pdf]

Supplementary material

Table S1. Selected genetic variants in serotonin system genes and allele frequencies

Gene	Chromosomal location	Variant	Allele	Allelic frequency	Population	Reference
TPH2	12q21.1	rs11178998	A	0.929	Non-Finnish European	gnomAD exomes v4.1 [1]
			G	0.071		
		rs4290270	T	0.645		
			A	0.355		
		rs7305115	G	0.574		
			A	0.426		
SLC6A4	17q11.2	5-HTTLPR[1]	L	0.586	European[2]	[2]
			S	0.414		
MAOA	Xp11.3	uVNTR[3]	3R	0.331	Caucasian American non-Hispanic; Caucasian European	[3, 4]
			3.5R	0.005		
			4R	0.648		
			5R	0.016		

[1]5-HT transporter linked polymorphic region in the *SLC6A4* gene. L represents the long (major) allele, and S the short (minor) allele.

[2] – Including: Croatia, Russia, Germany, Austria, United Kingdom, Hungary, Italy and Spain.

[3]uVNTR - upstream variable number tandem repeat alleles in the promoter of the *MAOA* gene. In this locus, we observed alleles with 3, 3.5, 4, and 5 repeats (3R, 3.5R, 4R, and 5R, respectively).

Table S2: Allele and genotype frequencies in *TPH2*, *SLC6A4*, and *MAOA* genetic variants in patients with bipolar disorder, major depressive disorder, schizophrenia and unaffected individuals.

				BD n = 101		MDD n = 148		SCH n = 143		Total n = 392		Unaffected individuals
Gene	Genetic variant			SA n = 46	non-SA n = 55	SA n = 76	non-SA n = 72	SA n = 53	non-SA n = 90	SA n = 175	non-SA n = 217	n = 140
TPH2	rs11178998	Genotype	A/A	0.91	0.93	0.76	0.83	0.81	0.86	0.82	0.87	0.90
			A/G	0.09	0.07	0.24	0.17	0.19	0.13	0.18	0.13	0.10
			G/G	0.00	0.00	0.00	0.00	0.00	0.01	0.00	0.00	0.00
		Allele	A	0.96	0.96	0.88	0.92	0.91	0.92	0.91	0.93	0.95
			G	0.04	0.04	0.12	0.08	0.09	0.08	0.09	0.07	0.05
	rs4290270	Genotype	A/A	0.46	0.44	0.50	0.49	0.47	0.47	0.48	0.47	0.11
			A/T	0.41	0.33	0.38	0.40	0.40	0.38	0.39	0.37	0.52
			T/T	0.13	0.24	0.12	0.11	0.13	0.16	0.13	0.16	0.36
		Allele	A	0.66	0.60	0.69	0.69	0.67	0.66	0.68	0.65	0.38
			T	0.34	0.40	0.31	0.31	0.33	0.34	0.32	0.35	0.62
	rs7305115	Genotype	A/A	0.50	0.36	0.50	0.56	0.43	0.52	0.48	0.49	0.15
			A/G	0.35	0.33	0.33	0.29	0.42	0.33	0.36	0.32	0.50
			G/G	0.15	0.31	0.17	0.15	0.15	0.14	0.16	0.19	0.35
		Allele	A	0.67	0.53	0.66	0.70	0.64	0.69	0.66	0.65	0.40
			G	0.33	0.47	0.34	0.30	0.36	0.31	0.34	0.35	0.60
SLC6A4	5-HTTLPR[1]	Genotype	L/L	0.43	0.51	0.46	0.42	0.53	0.48	0.47	0.47	0.33
			L/S	0.37	0.27	0.30	0.42	0.36	0.34	0.34	0.35	0.50
			S/S	0.20	0.22	0.24	0.17	0.11	0.18	0.19	0.18	0.17
		Allele	L	0.62	0.65	0.61	0.63	0.71	0.65	0.64	0.64	0.58
			S	0.38	0.35	0.39	0.38	0.29	0.35	0.36	0.36	0.42
MAOA[2]	uVNTR[3]	Genotype	3R/3R	0.15	0.11	0.08	0.08	0.07	0.03	0.10	0.07	0.04
			3R/4R	0.23	0.34	0.35	0.23	0.30	0.27	0.31	0.28	0.28
			3R/5R	0.02	0.02	0.00	0.01	0.00	0.00	0.01	0.01	0.01
			3R/3.5R	0.00	0.00	0.00	0.00	0.00	0.02	0.00	0.01	0.00
			3.5R/4R	0.00	0.02	0.00	0.00	0.00	0.00	0.00	0.00	0.01
			4R/4R	0.32	0.27	0.29	0.25	0.17	0.25	0.26	0.26	0.31
			4R/5R	0.00	0.02	0.00	0.03	0.00	0.00	0.00	0.01	0.01

			3R*	0.17	0.05	0.09	0.18	0.09	0.10	0.11	0.11	0.13
			4R*	0.06	0.16	0.18	0.18	0.35	0.31	0.20	0.23	0.21
			5R*	0.02	0.00	0.00	0.03	0.00	0.00	0.01	0.01	0.00
		Allele	3R	0.43	0.36	0.35	0.37	0.35	0.29	0.37	0.34	0.30
			3.5R	0.00	0.01	0.00	0.00	0.00	0.01	0.00	0.01	0.00
			4R	0.55	0.61	0.65	0.59	0.65	0.70	0.62	0.60	0.69
			5R	0.02	0.02	0.00	0.04	0.00	0.00	0.01	0.04	0.01

BD – bipolar disorder; MDD – major depressive disorder; SCH – schizophrenia; SA – patients with a history of suicide attempt; non-SA – patients without a history of suicide attempt.

[1]5-HT transporter linked polymorphic region in the *SLC6A4* gene. L represents the long (major) allele, and S the short (minor) allele.

[2]Given that *MAOA* gene is located on the X chromosome, the genotype column for *MAOA* includes genotypes for both males and females. Male genotypes are denoted with an asterisk (*) symbol.

[3]uVNTR - upstream variable number tandem repeat alleles in the promoter of the *MAOA* gene. In this locus, we observed alleles with 3, 3.5, 4, and 5 repeats (3R, 3.5R, 4R, and 5R, respectively). Males are hemizygous but coded as homozygous.

All variants were found to be in Hardy-Weinberg equilibrium among the control group.

Genotype and allele frequency data for the BD patients and unaffected individuals were taken from Radenkovic et al. [21].

Table S3: Average values of model-derived statistical measures in patients with bipolar disorder, major depressive disorder, schizophrenia and unaffected individuals.

		BD n = 101		MDD n = 148		SCH n = 143		Unaffected individuals
Molecular species	Statistical measure	SA n = 46	non-SA n = 55	SA n = 76	non-SA n = 72	SA n = 53	non-SA n = 90	n = 140
5-HTP	mean	13.62 ± 0.2	13.64 ± 0.2	13.70 ± 0.3	13.67 ± 0.3	13.66 ± 0.3	13.65 ± 0.3	13.63 ± 0.2
	median	6.55 ± 0.1	6.55 ± 0.1	6.59 ± 0.2	6.58 ± 0.2	6.57 ± 0.1	6.57 ± 0.1	6.55 ± 0.10
	maximum	42.23 ± 0.6	42.27 ± 0.6	42.47 ± 0.9	42.37 ± 0.8	42.33 ± 0.8	42.30 ± 0.8	42.24 ± 0.7
	standard deviation	14.58 ± 0.2	14.59 ± 0.2	14.66 ± 0.3	14.63 ± 0.3	14.62 ± 0.3	14.60 ± 0.3	14.58 ± 0.2
	skewness	0.79 ± 0.0	0.79 ± 0.0	0.79 ± 0.0	0.79 ± 0.0	0.79 ± 0.0	0.79 ± 0.0	0.79 ± 0.0
	kurtosis	2.09 ± 0.0	2.09 ± 0.0	2.09 ± 0.0	2.09 ± 0.0	2.09 ± 0.0	2.09 ± 0.0	2.09 ± 0.0
fc5-HT	mean	0.94 ± 0.3	0.85 ± 0.2	0.87 ± 0.3	0.93 ± 0.3	0.90 ± 0.3	0.89 ± 0.3	0.89 ± 0.3
	median	0.61 ± 0.2	0.54 ± 0.2	0.56 ± 0.2	0.60 ± 0.2	0.58 ± 0.2	0.57 ± 0.2	0.57 ± 0.2
	maximum	2.53 ± 0.7	2.31 ± 0.6	2.36 ± 0.6	2.51 ± 0.7	2.43 ± 0.6	2.40 ± 0.6	2.40 ± 0.6
	standard deviation	0.88 ± 0.2	0.80 ± 0.2	0.82 ± 0.2	0.87 ± 0.2	0.84 ± 0.2	0.83 ± 0.2	0.83 ± 0.2
	skewness	0.60 ± 0.0	0.61 ± 0.0	0.61 ± 0.0	0.60 ± 0.0	0.60 ± 0.0	0.61 ± 0.0	0.61 ± 0.0
	kurtosis	1.84 ± 0.0	1.85 ± 0.0	1.85 ± 0.0	1.84 ± 0.0	1.84 ± 0.0	1.85 ± 0.0	1.85 ± 0.0
v5-HT	mean	2.63 ± 0.7	2.4 ± 0.6	2.45 ± 0.7	2.61 ± 0.7	2.52 ± 0.7	2.50 ± 0.7	2.49 ± 0.7
	median	1.81 ± 0.6	1.61 ± 0.6	1.66 ± 0.6	1.79 ± 0.6	1.72 ± 0.6	1.70 ± 0.6	1.69 ± 0.6
	maximum	6.81 ± 1.6	6.29 ± 1.4	6.40 ± 1.5	6.76 ± 1.5	6.56 ± 1.5	6.50 ± 1.5	6.49 ± 1.5
	standard deviation	2.37 ± 0.5	2.19 ± 0.5	2.23 ± 0.5	2.35 ± 0.5	2.28 ± 0.5	2.27 ± 0.5	2.26 ± 0.5
	skewness	0.54 ± 0.1	0.55 ± 0.1	0.55 ± 0.1	0.54 ± 0.1	0.54 ± 0.1	0.55 ± 0.1	0.55 ± 0.1
	kurtosis	1.76 ± 0.0	1.78 ± 0.0	1.77 ± 0.0	1.76 ± 0.0	1.77 ± 0.0	1.77 ± 0.0	1.77 ± 0.0
e5-HT	mean	0.07 ± 0.0	0.07 ± 0.0	0.07 ± 0.0	0.07 ± 0.0	0.07 ± 0.0	0.07 ± 0.0	0.07 ± 0.0
	median	0.04 ± 0.0	0.04 ± 0.0	0.04 ± 0.0	0.04 ± 0.0	0.04 ± 0.0	0.04 ± 0.0	0.04 ± 0.0
	maximum	0.20 ± 0.0	0.20 ± 0.0	0.20 ± 0.0	0.20 ± 0.0	0.20 ± 0.0	0.20 ± 0.0	0.20 ± 0.0
	standard deviation	0.07 ± 0.0	0.07 ± 0.0	0.07 ± 0.0	0.07 ± 0.0	0.07 ± 0.0	0.07 ± 0.0	0.07 ± 0.0
	skewness	0.64 ± 0.0	0.65 ± 0.0	0.65 ± 0.0	0.64 ± 0.0	0.65 ± 0.0	0.65 ± 0.0	0.65 ± 0.0
	kurtosis	1.89 ± 0.0	1.90 ± 0.0	1.90 ± 0.0	1.89 ± 0.0	1.90 ± 0.0	1.90 ± 0.0	1.90 ± 0.0
5-HIAA	mean	0.50 ± 0.3	0.59 ± 0.2	0.59 ± 0.2	0.53 ± 0.3	0.61 ± 0.2	0.63 ± 0.2	0.61 ± 0.2
	median	0.52 ± 0.3	0.61 ± 0.2	0.62 ± 0.3	0.56 ± 0.3	0.63 ± 0.3	0.66 ± 0.2	0.63 ± 0.3

maximum	0.82 ± 0.5	0.97 ± 0.4	0.98 ± 0.4	0.88 ± 0.5	1.01 ± 0.4	1.05 ± 0.4	1.00 ± 0.4
standard deviation	0.25 ± 0.1	0.29 ± 0.1	0.29 ± 0.1	0.26 ± 0.1	0.30 ± 0.1	0.31 ± 0.1	0.30 ± 0.1
skewness	-0.41 ± 0.0	-0.4 ± 0.0	-0.40 ± 0.0	-0.41 ± 0.0	-0.40 ± 0.0	-0.40 ± 0.0	-0.40 ± 0.0
kurtosis	2.11 ± 0.0	2.1 ± 0.0	2.11 ± 0.0	2.11 ± 0.0	2.11 ± 0.0	2.11 ± 0.0	2.11 ± 0.0

BD – bipolar disorder; MDD – major depressive disorder; SCH – schizophrenia; SA – patients with a history of suicide attempt; non-SA – patients without a history of suicide attempt.

5-HTP – 5-hydroxytryptophan; fc5-HT – free cellular serotonin (5-HT); v5-HT – vesicular 5-HT; e5-HT – extracellular 5-HT;

5-HIAA – 5-hydroxyindoleacetic acid.

Table S4: Aligned Rank Transform (ART) ANOVA results for all model-derived features across diagnostic and suicide attempt groups.

		Suicide attempt		Diagnosis		Suicide attempt x Diagnosis	
Molecular species	Feature	F	p-value	F	p-value	F	p-value
5-HTP	mean	0.14	0.706	1.79	0.168	0.19	0.826
	median	0.22	0.638	1.44	0.239	0.22	0.799
	maximum	0.14	0.706	1.79	0.168	0.18	0.834
	standard deviation	0.14	0.706	1.79	0.168	0.19	0.826
	skewness	0.14	0.708	1.81	0.166	0.20	0.820
	kurtosis	0.14	0.707	1.79	0.168	0.20	0.820
fc5-HT	mean	1.14	0.286	0.41	0.667	2.76	0.064[1]
	median	1.02	0.314	0.54	0.584	2.49	0.084[1]
	maximum	0.95	0.331	0.43	0.648	2.88	0.057[1]
	standard deviation	0.89	0.346	0.43	0.648	2.75	0.065[1]
	skewness	0.79	0.374	0.41	0.665	1.59	0.206
	kurtosis	0.61	0.435	0.50	0.607	1.45	0.236
v5-HT	mean	1.04	0.308	0.44	0.644	2.72	0.067[1]
	median	1.29	0.257	0.42	0.658	2.09	0.125
	maximum	0.84	0.361	0.45	0.639	2.94	0.054[1]
	standard deviation	0.85	0.356	0.48	0.621	2.79	0.063[1]
	skewness	0.92	0.339	0.47	0.623	1.99	0.138
	kurtosis	0.54	0.465	0.43	0.653	1.87	0.156
e5-HT	mean	0.58	0.446	2.31	0.100	0.23	0.798
	median	0.07	0.789	1.06	0.349	2.46	0.087[1]
	maximum	0.42	0.517	1.71	0.183	0.39	0.679
	standard deviation	0.26	0.612	1.16	0.316	0.56	0.572
	skewness	0.09	0.768	0.73	0.483	1.68	0.188
	kurtosis	0.01	0.939	0.86	0.423	1.63	0.198
5-HIAA	mean	0.12	0.727	4.61	**0.011**	2.46	0.087[1]
	median	0.07	0.788	4.54	**0.011**	2.25	0.107
	maximum	0.15	0.699	4.62	**0.010**	2.62	0.074[1]
	standard deviation	0.10	0.748	4.49	**0.012**	2.25	0.107
	skewness	2.31	0.130	0.40	0.672	4.38	**0.013**
	kurtosis	1.02	0.312	0.45	0.641	2.33	0.099[1]

[1] – Statistical trend.

5-HTP – 5-hydroxytryptophan; fc5-HT – free cellular serotonin (5-HT); v5-HT – vesicular 5-HT; e5-HT – extracellular 5-HT; 5-HIAA – 5-hydroxyindoleacetic acid.

Statistically significant results are shown in bold.

References

1. Chen S, Francioli LC, Goodrich JK, et al. A genomic mutational constraint map using variation in 76,156 human genomes. Nature. 2024;625(7993):92-100. doi:10.1038/s41586-023-06045-0
2. Noskova T, Pivac N, Nedic G, et al. Ethnic differences in the serotonin transporter polymorphism (5-HTTLPR) in several European populations. Prog Neuropsychopharmacol Biol Psychiatry. 2008;32(7):1735-1739. doi:10.1016/j.pnpbp.2008.07.012
3. Sabol SZ, Hu S, Hamer D. A functional polymorphism in the monoamine oxidase A gene promoter. *Hum Genet*. 1998;103(3):273-279. doi:10.1007/s004390050816
4. Samochowiec A, Chęć M, Kopaczewska E, et al. Monoamine oxidase a promoter variable number of tandem repeats (MAOA-uVNTR) in alcoholics according to Lesch typology. *Int J Environ Res Public Health*. 2015;12(3):3317-3326. Published 2015 Mar 19. doi:10.3390/ijerph120303317
